# A Practical Approach to the Diagnosis of Pelvic Inflammatory Disease

**DOI:** 10.1155/2011/753037

**Published:** 2011-07-26

**Authors:** Oluwatosin Jaiyeoba, David E. Soper

**Affiliations:** Department of Obstetrics and Gynecology, Medical University of South Carolina, P.O. Box 250619, Charleston, SC 29425, USA

## Abstract

The diagnosis of acute pelvic inflammatory disease (PID) is usually based on clinical criteria and can be challenging for even the most astute clinicians. Although diagnostic accuracy is advocated, antibiotic treatment should be instituted if there is a diagnosis of cervicitis or suspicion of acute PID. Currently, no single test or combination of diagnostic indicators have been found to reliably predict PID, and laparoscopy cannot be recommended as a first line tool for PID diagnosis. For this reason, the clinician is left with maintaining a high index of suspicion for the diagnosis as he/she evaluates the lower genital tract for inflammation and the pelvic organs for tenderness in women with genital tract symptoms and a risk for sexually transmitted infection. This approach should minimize treating women without PID with antibiotics and optimize the diagnosis in a practical and cost-effective way.

## 1. Introduction

Acute PID is associated with significant sequelae including tubal factor infertility, ectopic pregnancy, and chronic pelvic pain. To ameliorate these adverse outcomes, an approach to its diagnosis must promote the ability to intervene with antimicrobial therapy early on the course of this ascending infection. It is less important to accurately determine where the patient may lie along the continuum of this ascending inflammatory process (cervicitis, endometritis, salpingitis, or peritonitis) and more important to empirically initiate an appropriate antibiotic regimen when the diagnosis is suspected.

There is a wide variation in the symptoms, some of which fail to imply a pelvic etiology, associated with acute PID (Table 1). They may range from subtle or mild to severe. This requires the clinician to maintain a high index of suspicion for the diagnosis of PID. Alternatively, the signs of PID are limited to an inflammatory exudate from the lower genital tract and pelvic organ tenderness. The value of recognizing the symptoms associated with acute PID is based on their ability to trigger the clinician's evaluation of the pelvis. If pelvic examination fails to reveal evidence of inflammation (if there is no leukorrhea), then the diagnosis of PID is much less likely and antibiotic treatment can be withheld while the remaining diagnostic workup defines the diagnosis. However, evidence of lower genital tract inflammation and any pelvic organ tenderness suggests the advisability of initiating antimicrobial therapy for a diagnosis of PID. 

Laparoscopy can confirm the presence of acute salpingitis in a patient with a clinical diagnosis of PID. However, laparoscopy cannot be used to dictate which patients are candidates for antimicrobial therapy as women without acute salpingitis still require antimicrobial therapy for a clinical diagnosis of endometritis without salpingitis. Therefore, despite laparoscopy being the gold standard for the diagnosis of acute salpingitis, its routine use is neither feasible nor recommended. 

The clinical diagnosis of PID is imprecise. Most studies confirm the positive predictive value (PPV) of a clinical diagnosis of PID for salpingitis of 65% when confirmed by laparoscopy. No single historical, physical, or laboratory finding is reliably diagnostic for acute PID. We are therefore left with the challenge of diagnosing PID in such a way as to minimize its associated sequelae while at the same time not over treating all women with pelvic pain or other genital tract symptoms with antimicrobials.

## 2. Challenges

1. Determine which women presenting with genital tract symptoms are candidates for antibiotic therapy for a diagnosis of acute PID.
2. Determine which women with acute PID actually have acute salpingitis since these women are at the highest risk for the reproductive sequelae associated with this disease.

## 3. Composite Clinical Criteria

The diagnosis of PID should be considered in all sexually active women with or without lower abdominal pain and symptoms noted in Table 1. An assessment of risk for sexually transmitted infection (STI) enhances the specificity of these presenting symptoms. However, women without such risk factors should still have the diagnosis considered given that many will not be accurate in believing that they reside in a mutually monogamous sexual relationship [1]. Abdominal tenderness may not be present in many women with PID, particularly if peritonitis is not present or the patient has endometritis without salpingitis. A bimanual pelvic examination may reveal pelvic organ tenderness, uterine tenderness in the case of endometritis, and adnexal tenderness in the case of salpingitis. Cervical motion tenderness is another common finding in women with PID. The Centers for Disease Control and Prevention (CDC) [2] recommend empiric treatment for PID in sexually active young women (25 years old or younger) and other women at risk of STI (multiple sex partners or history of STI) if they are experiencing pelvic or lower abdominal pain, if no cause for the illness other than PID can be identified, and if one or more of the following is appreciated on bimanual pelvic examination: cervical motion tenderness, uterine tenderness, or adnexal tenderness. The limitation of this approach is that it fails to discriminate between the differential diagnoses of acute pelvic pain in reproductive-aged women. For this reason, the lower genital tract needs to be assessed for signs of inflammation. The cervical canal should be examined for the presence of yellow or green mucopus and friability. Microscopy of the vaginal secretions should be performed looking for leukorrhea (more than 1 leukocyte per epithelial cell). Evaluation for bacterial vaginosis (vaginal pH, clue cells, and whiff test) and trichomonas vaginitis is in order [3–6]. Finally, nucleic acid amplification testing (NAAT) for *Neisseria gonorrhoeae *and* Chlamydia trachomatis *should be performed. If the cervix is normal and no white blood cells are noted during microscopy of the vaginal secretions, an alternative diagnosis should be investigated since this reliably excludes (negative predictive value 94.5%) upper genital tract infection [7]. Because the sensitivity of microscopy to detect *Trichomonas vaginalis* is relatively low (approximately 50%), symptomatic women with cervicitis and negative microscopy for trichomonads should receive further testing (i.e., culture or polymerase chain reaction method). Standardized diagnostic tests for *Mycoplasma genitalium* are not routinely performed. Empiric antibiotic treatment should be initiated in sexually active young women, especially those at risk for sexually transmitted infections (STIs), with pelvic or lower abdominal pain, if no other causes other than PID can be identified and if the following minimum criteria are present on pelvic examination:

- lower genital tract inflammation (cervicitis and/or leukorrhea (>1 leukocyte per epithelial cell on microscopy of the vaginal secretions))
- any pelvic organ tenderness (e.g., cervical motion tenderness, uterine tenderness, or adnexal tenderness)

The above approach is sufficient to assure that women with PID will be treated appropriately with antibiotics. At least a third of these women will not have acute salpingitis, but never the less are candidates for antibiotic therapy. Given that antibiotic regimens are identical for the treatment of women with acute salpingitis regardless of degree of severity, there is no utility in confirming the diagnosis laparoscopically. 

If women with the clinical diagnosis of PID were to undergo routine laparoscopy, visual evidence of acute tubal inflammation (erythema, edema, and purulent exudate) would be confirmed approximately 65% of the time [8, 9]. Therefore, the clinical diagnosis of PID may represent women with visually confirmed acute salpingitis. However, the clinical diagnosis of PID may also represent women with cervicitis and endometritis without salpingitis or with cervicitis alone [10, 11]. *C. trachomatis, N. gonorrhoeae*, bacterial vaginosis, and trichomonas vaginitis are associated with histologic evidence of endometritis in women without the clinical manifestations of PID [10]. The symptoms and signs of PID are essentially indistinguishable among women with acute salpingitis, those with endometritis without acute salpingitis, and those with cervicitis but neither endometritis nor salpingitis [11–13]. 

Other ancillary tests (Table 2) that can be useful in diagnosing PID include a complete blood count, erythrocyte sedimentation rate (ESR), or C-reactive protein (CRP). These tests are recommended for patients with clinically severe PID. Imaging studies are most helpful when ruling out competing differential diagnoses such as the use of pelvic ultrasonography to rule out symptomatic ovarian cysts and computed tomography to rule out appendicitis. Pelvic ultrasonography has limited sensitivity for the diagnosis of PID, but the specific finding of thickened fluid-filled tubes by ultrasonography supports the diagnosis of upper genital tract inflammation [14]. Pelvic ultrasonography should be ordered in patients requiring hospitalization or those with a pelvic mass.

## 4. Laboratory Tests

White blood cell (WBC) counts are beneficial when abnormal. However, only 60% of patients with PID present with elevated serum WBC count [15]. ESR, a nonspecific inflammatory marker has been found to be elevated in PID but an elevated ESR (>15 mm/h) is only present in approximately 75% of women with acute PID and, as a nonspecific maker of inflammation, can be found in other disease states. CRP, another inflammatory marker, has been studied in acute PID. In a series that involved 152 patients, a CRP >10 mg/dL had a good sensitivity (93%) and specificity (83%) in the diagnosis of PID [16]. Furthermore, CRP levels decrease to normal sooner than ESR following effective antibiotic therapy and may be beneficial as a monitoring tool.

There might be a role for CA-125 in PID diagnosis. Duk et al. from The Netherlands looked at the relationship of CA-125 in 50 patients with a provisional diagnosis of PID and concluded that the finding of an elevated serum CA-125 level confirms the diagnosis of peritoneal involvement in patients with a clinical diagnosis of PID [17]. They measured CA-125 concentrations in serum before laparoscopy and during hospitalization, using an enzyme immunoassay and found that CA-125 concentration before laparoscopy correlated with the extent of inflammatory peritoneal involvement and the predictive value of an elevated serum CA-125 level to indicate the presence of salpingitis (grades 1–3) was 97%. However, the predictive value of a normal CA-125 level indicating normal observations at laparoscopy (grade 0) was only 47%. Similarly, Mozas and coworkers [18] in Spain looked at the efficiency of different tumor markers (CA-125, carcinoembryonic antigen, CA-15.3, CA-19.9) and insulin-like growth factor I (IGF-I) measurements as a screening procedure for acute PID, and found no differences in the levels of CA-15.3, CA-19.9, carcinoembryonic antigen and IGF-I between three groups studied. However, the serum levels of CA-125 were significantly higher in patients who had PID and they concluded that measurement of serum CA-125 concentrations is recommended as a useful test for acute PID in patients undergoing laparoscopy for pelvic pain. Paavonen and coworkers in Finland measured serum levels of CA-125 in 31 patients with confirmed PID and found a correlation between CA-125 levels and the severity of adnexal inflammation as defined by laparoscopy. There was no association between isolation of specific microorganisms from the upper genital tract and elevated CA-125, and in most of the women in this study, serum levels of CA-125 decreased during treatment [19]. Finally, Moore and Soper [20] also reported a relationship between CA-125 and laparoscopically confirmed acute salpingitis and further noted that the degree of elevation of CA-125 levels correlated with severity of tubal inflammation.

## 5. Endometrial Biopsy

Endometrial biopsy has been studied extensively in the diagnosis of PID. It is less invasive compared with laparoscopy. The presence of neutrophils and plasma cells in the endometrium is indicative of endometritis and may be used to diagnose PID [21].

Kiviat and coworkers [22] looked at endometrial histopathology in 69 patients with clinically suspected acute PID and reported that 54% of the patients had both upper genital tract infection (UGTI) and laparoscopically confirmed salpingitis. They reported UGTI without salpingitis in 1%, while salpingitis without UGTI was reported in 16%. The study found that the simultaneous presence of five or more neutrophils per ×400 field in endometrial surface epithelium, together with one or more plasma cells per ×120 field in endometrial stroma were the best predictor of upper genital tract infection plus salpingitis. This combination had a sensitivity of 92% and a specificity of 87% for predicting the diagnosis of both UGTI and laparoscopically confirmed acute salpingitis. Additionally, 90% of all UGTIs identified in this study were attributable to *C. trachomatis *or* N. gonorrhoeae,* and 92% of the women diagnosed with UGTI and salpingitis had either chlamydia or gonorrhea infection.

## 6. Imaging Studies

Ultrasonography is the imaging method of choice, followed closely by Magnetic Resonance Imaging (MRI). Computed tomography (CT) is reserved for evaluation of the extent of PID within the abdomen and interventional management. Ultrasonography is noninvasive, widely available, and a good diagnostic tool to have in a physician's armamentarium for PID diagnosis. The typical ultrasound findings in acute PID have been described by Timor-Tritsch and Rottem [14], and the addition of Power Doppler to transvaginal ultrasonography has been found to increase its sensitive in diagnosis of PID. Transvaginal ultrasonography is preferred to transabdominal approach and also helpful in guiding needles to drain abscesses. MRI is expensive but more sensitive. Tukeva et al. [23] compared transvaginal ultrasonography, MRI, and laparoscopy in 30 in-patients hospitalized in Finland with clinically suspected PID and reported that MRI diagnoses were 95% correct in 21 women with laparoscopically acute salpingitis compared with transvaginal sonogram that was 81% accurate. The sensitivity of MRI in the diagnosis of PID was found to be 95%, with a specificity of 89%, and overall accuracy was 93%. For transvaginal US, the corresponding value was 81%, 78%, and 80%, respectively. MRI is more accurate than transvaginal US and provides information about the differential diagnosis of PID, and as such its use may also reduce the need for diagnostic laparoscopy.

Although the literature is replete with reports regarding the sonographic findings of PID, little was published about CT images until the last decade [24]. CT findings in early PID include obscuration of the normal pelvic floor fascial planes, thickening of the uterosacral ligaments, cervicitis, oophoritis, salpingitis, and accumulation of simple fluid in the endometrial canal, fallopian tubes, and pelvis. The simple fluid may become complex as the disease progresses and eventually become a frank tuboovarian or pelvic abscess. Reactive inflammation can manifest as small or large bowel ileus or obstruction, hydronephrosis or hydroureter, and right upper quadrant inflammation (Fitz-Hugh-Curtis syndrome). One drawback of CT images however is the exposure to ionizing radiation, which can be problematic in young women.

 If imaging is considered, we would first recommend transvaginal ultrasound, and if classic findings of PID are noted on ultrasound [14], no further imaging is required. If additional characterization is warranted, then we recommend MRI over CT because its overall accuracy is greater than 93% and does not carry the additional risk of ionizing radiation. If tuboovarian abscess (TOA) is suspected, we recommend an initial transvaginal ultrasound because this is the most cost effective imaging to allow percutaneous drain placement. However, many interventional radiologists will prefer CT to guide drain placement.

## 7. Laparoscopy

Laparoscopy has been shown to add considerable accuracy to the clinical methods of diagnosing acute salpingitis [9]. The procedure does not aggravate the inflammatory process. Jacobson and Weström looked at 905 cases over an eight-year period (1960–1967) and set the standard for laparoscopic diagnosis. The minimum laparoscopic criteria for visual diagnosis of acute salpingitis include: pronounced hyperemia of the tubal surface, edema of the tubal wall, and, thirdly, a sticky exudate on the tubal surface and from the fimbriated ends when patent. In their study, they hardly encountered difficulties differentiating mild pathologic changes and normal conditions, but one major drawback that can be envisaged is the patient with endometritis who has no salpingitis. In 814 cases in their series with suspected acute PID, 532 (65%) had laparoscopically confirmed acute salpingitis, 12% had other pathologic conditions, and 23% had no pathologic conditions changes. We suspect that a significant proportion of women in the latter category had endometritis without salpingitis. 

In another study comparing clinical and laboratory findings with laparoscopic findings of acute PID, Eschenbach and coworkers [25] reported that the severity of clinical and laboratory manifestations (other than adnexal mass) was not associated positively with tubal occlusion and that the severity of some findings was actually associated negatively with the severity of tubal damage.

Although laparoscopy is referred to as the “gold standard” for the diagnosis of PID, review of the literature regarding its accuracy has been mixed. Accuracy of a clinical diagnosis when compared with diagnostic laparoscopy in the diagnosis of PID has been reported in various studies. Morcos et al. [26] in a study of 176 women with clinically diagnosed PID established laparoscopically confirmed PID in 76.1% of the cohort. Similarly, Cohen et al. [21] in a prospective case-control study investigated the etiology of acute salpingitis in a cohort of Kenyan women and confirmed salpingitis laparoscopically in 142 (90%) of the 158 women with a clinical diagnosis of acute PID. Conversely, Peipert et al. [27] found the sensitivities of both the accepted clinical criteria and the triad of laparoscopy visualization of edema, erythema and purulent exudates to be low. In that study, the sensitivities of the CDC's minimal clinical criteria for PID and the laparoscopic triad of edema, erythema, and purulent exudates were 65% and 60%, respectively. Sellors et al. [28] also found laparoscopy to be 50% and 80% sensitive and specific, respectively. In this study, additional evidence from endometrial and fimbrial biopsy increased the prevalence of confirmed PID from 30% in visual diagnosis alone to 46% when endometrial and fimbrial minibiopsy evidence was included.

Women with a recurrent diagnosis of PID and persistently negative NAATs and who are classified as “lower risk” epidemiologically should have laparoscopy to consider alternative diagnoses such as endometriosis.

## 8. Conclusion

Diagnostic laparoscopy with concomitant endometrial biopsy (subsequently examined histologically) in women with cervicitis will accurately define the continuum of inflammation associated with a clinical diagnosis of PID. This approach will allow the clinician/investigator to define as to whether the patient/subject has cervicitis/endometritis/acute salpingitis, cervicitis/endometritis, or cervicitis alone. This comprehensive approach is neither practical nor cost effective for those not in a research setting.

**Figure 1:**
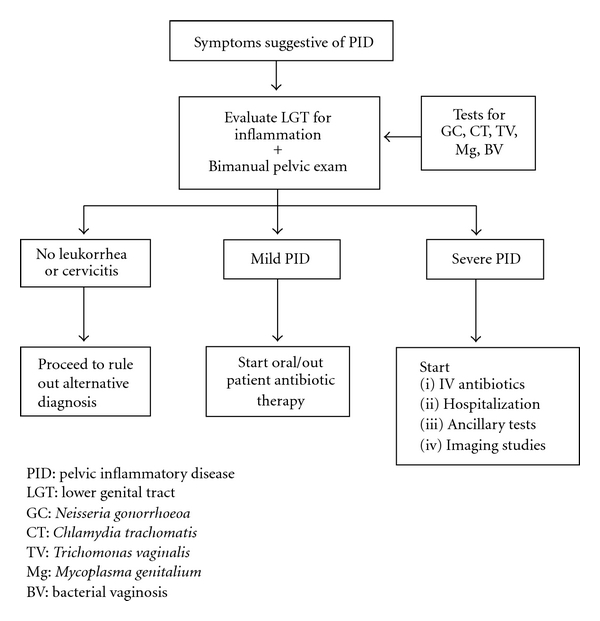
Flow chart Showing Clinical Diagnosis of PID.

A purely clinical approach using the findings of lower genital tract inflammation (leukorrhea) associated with pelvic organ tenderness will identify the vast majority of women with PID, and all are candidates for antibiotic therapy (Figure 1). We recommend this approach as the most practical and cost effective. 

Finally, additional testing and imaging is important in two scenarios first, in differentiating alternative diagnoses such as ovarian cysts and appendicitis. Second, in the more seriously ill patient who needs additional evaluation to assess the degree of sepsis and to consider the presence of a tuboovarian abscess. Women with severe PID are candidates for hospital admission and parenteral antibiotic therapy.

## Figures and Tables

**Table 1:** Symptoms in women with clinically suspected pelvic inflammatory disease.

Abdominal pain	
Abnormal discharge	
Intermenstrual bleeding	
Postcoital bleeding	
Fever	
Urinary frequency	
Low back pain	
Nausea/vomiting	

Data from [5].

**Table 2:** Signs and tests to increase the specificity of a diagnosis of salpingitis.

An additional sign and abnormal laboratory tests increase the specificity of the diagnosis of PID:	
(i) Oral temperature >101 F (>38.3°C)	
(ii) Elevated C-reactive protein (CRP)	
(iii) Laboratory documentation of cervical *Neisseria gonorrhoeae* or *Chlamydia trachomatis*.	
The most specific criteria for diagnosis of PID include:	
(i) Endometrial biopsy with histologic evidence of endometritis	
(ii) Transvaginal sonography or MRI showing thickened, fluid-filled tubes with or without free pelvic or tuboovarian complex or dopplers studies suggesting pelvic infection (tubal hyperemia)	
(iii) Laparoscopic abnormalities consistent with PID	

Data from [2].
